# Risk Factors for Radiation Necrosis in Patients Undergoing Cranial Stereotactic Radiosurgery

**DOI:** 10.3390/cancers13194736

**Published:** 2021-09-22

**Authors:** Johannes Kerschbaumer, Matthias Demetz, Aleksandrs Krigers, Meinhard Nevinny-Stickel, Claudius Thomé, Christian F. Freyschlag

**Affiliations:** 1 Department of Neurosurgery, Medical University of Innsbruck, 6020 Innsbruck, Austria; matthias.demetz@student.i-med.ac.at (M.D.); aleksandrs.krigers@tirol-kliniken.at (A.K.); claudius.thome@tirol-kliniken.at (C.T.); christian.freyschlag@tirol-kliniken.at (C.F.F.); 2 Department of Radiotherapy and Radiation Oncology, Medical University of Innsbruck, 6020 Innsbruck, Austria; meinhard.nevinny-stickel@tirol-kliniken.at

**Keywords:** radiotherapy, stereotactic radiosurgery, SRS, radiation necrosis, brain metastasis, meningioma, acoustic neuroma, tumor diameter, radiation dose, risk factors

## Abstract

**Simple Summary:**

Radiation necrosis is a known complication after stereotactic radiosurgery of intracranial tumors. We evaluated 388 patients who underwent stereotactic radiosurgery at our institution. The most common tumors were metastases (47.2%), followed by vestibular schwannomas (32.2%) and meningiomas (13.4%). 15.7% developed radiation necrosis after a median of 8 months. According to our data, larger tumor diameter (HR 1.065) and higher radiation dose (HR 1.302) were associated with an increased risk of radiation necrosis independently of tumor type. Advanced age was shown to be a risk factor for radiation necrosis only in cases with metastasis (HR 1.066). The data from this study suggest that the development of radiation necrosis is dependent on size and dose, not on the type of the neoplasm.

**Abstract:**

Purpose: single-staged stereotactic radiosurgery (SRS) is an established part of the multimodal treatment in neuro-oncology. Radiation necrosis after high-dose irradiation is a known complication, but there is a lack of evidence about the risk factors. The aim of this study was to evaluate possible risk factors for radiation necrosis in patients undergoing radiosurgery. Methods: patients treated with radiosurgery between January 2004 and November 2020 were retrospectively analyzed. The clinical data, imaging and medication were gathered from electronic patient records. The largest diameter of the tumors was measured using MRI scans in T1 weighted imaging with gadolinium and the edema in T2 weighted sequences. The diagnosis of a radiation necrosis was established analyzing imaging criteria combined with clinical course or pathologically confirmed by subsequent surgical intervention. Patients developing radiation necrosis detected after SRS were compared to patients without evidence of an overshooting irradiation reaction. Results: 388 patients were included retrospectively, 61 (15.7%) of whom developed a radiation necrosis. Median follow-up was 24 (6–62) months with a radiation necrosis after 8 (6–12) months. The most frequent tumors were metastases in 47.2% of the cases, followed by acoustic neuromas in 32.2% and meningiomas in 13.4%. Seventy-three (18.9%) patients already underwent one or more previous radiosurgical procedures for different lesions. The mean largest diameter of the tumors amounted to 16.3 mm (±6.1 mm). The median—80%—isodose administered was 16 (14–25) Gy. Of the radiation necroses, 25 (43.1%) required treatment, in 23 (39.7%) thereof, medical treatment was applied and in 2 (3.4%) cases, debulking surgery was performed. In this study, significantly more radiation necroses arose in patients with higher doses (HR 1.3 [CI 1.2; 1.5], *p* < 0.001) leading to a risk increment of over 180% between a radiation isodose of 14 and 20 Gy. The maximum diameter was a second significant risk factor (*p* = 0.028) with an HR of 1065 for every 1 mm increase in multivariate analysis. Conclusion: large diameter and high doses were reliable independent risk factors leading to more frequent radiation necroses, regardless of tumor type in patients undergoing radiosurgery. Alternative therapeutic procedures may be considered in lesions with large volume and an expected high radiation doses due to the increased risk of developing radiation necrosis.

## 1. Introduction

Stereotactic radiosurgery (SRS) plays an important role in multimodal neuro-oncological treatment of a broad spectrum of intracranial diseases. Additionally, the technique has been introduced as treatment option in trigeminal neuralgia [1], in case of inoperable arteriovenous malformations [2] and has also been used in the treatment of refractory epilepsy [3]. SRS is widely used for the treatment of metastases [4], meningiomas [5] and vestibular schwannomas (VS) [6,7] where long-term local tumor control can be achieved in VS and meningioma [8,9,10,11]. As a major advantage compared to conventional external beam radiotherapy, SRS aims to spare surrounding tissue.

Radiation necrosis (RN) can be a sequela of this technique due to the high doses achieved in the tumors and may lead to neurological deterioration with associated impairment in patient’s quality of life (QoL) [12]. Quoting the existing literature, RN develops with an incidence of 13–14% [13] mostly within the first year after SRS for brain metastases (BM). There is little evidence about risk factors, especially because the induced necrosis due to the high radiation doses applied is the mode of action for SRS and a clear differentiation between expected tissue destruction and overwhelming necrotic changes can be cumbersome. Mainly, symptomatic enlargement with corresponding symptoms and edematous or destructive changes around the previously treated pathology are defined as RN, but often only the clinical course over time confirms the diagnosis. The available treatment options consist of symptomatic treatment with corticosteroids or bevacizumab (BEV), whereas surgical intervention and resection of a RN is used only in selected cases when progressive disease is suspected. Surgical decompression of very large lesions [14] with significant mass effect may be beneficial. There is no causal treatment, steroids are effective at reducing the edema and symptoms. Recently, evidence for BEV was found with a positive effect in a randomized double-blind study [15].

It has to be mentioned, that the risk of developing a symptomatic RN cannot be reliably predicted despite the increased use of SRS. In BM, a larger treatment volume [12] and concurrent systemic therapy [16] have been accused of higher RN-rates. In meningioma cases, the volume and tumor location were associated with a higher incidence of RN. In VS, no special risk factors have been linked to RN.

With this study, we aimed to establish risk factors for the development of RN after SRS that apply to different tumor types.

## 2. Materials and Methods

All patients who underwent SRS for an intracranial neoplasm (BM, meningioma or VS) between January 2004 and November 2020 were collected retrospectively and included in this analysis. Clinical and epidemiological data were retrieved from the electronic patient charts. Radiological data such as basic diameter and the radiological course after SRS were collected from the radiological database. Tumor entity was determined as radiological diagnosis (including appearance on MRI or known malignant history) and the tumor size was measured as the largest diameter on axial T1 sequences with gadolinium contrast. The largest axial diameter of the neoplasm was determined with an accuracy of 0.1 mm. The surrounding edema was identified at its greatest extent, excluding the tumor itself. The edema as a radiological sign of RN in the course after SRS was assessed on T2 weighted sequences.

Radiosurgery was performed with a linear accelerator (SL25, ELEKTA, 6 MeV photon beams) adapted for stereotactic radiosurgery and endued with changeable cylindric collimators (3–30 mm). In all patients, gadolinium-enhanced T1-weighted MPRAGE volume MR scans were performed with a slice thickness of 1 mm and reconstruction in 3 dimensions one or two days before treatment. For the treatment the Brain LAB (BrainLAB AG, Feldkirchen, Germany) treatment planning system, BrainScan, and immobilization hardware were used. SRS was performed using an invasive stereotactic head ring. With the stereotactic head ring in place, a contrast-enhanced CT scan was performed using the stereotactic localizer. The patient’s entire head was scanned using 1–3 mm contiguous slices. On these CT scans and fused MR-images the planning target volume and the organs of risk were outlined. The dose was 12–28 Gy to the prescription isodose line (80%). High conformality of the treatment dose to the borders of the planning target volume was established by different combinations of number, span and weight of noncoplanar arcs. Every effort was made to achieve homogeneity in dose distribution across the planning target volume while keeping the dose to the organs of risk as low as possible. The conformity index and the heterogeneity index could only be determined in 117 patients out of the cohort. For the rest, given the retrospective data evaluation, the indices were not calculated. Patients were routinely administered steroids after SRS. Typically, patients were given 3 × 4 mg dexamethasone orally for 5 days followed by decreasing dosage of 2 mg every 5 days.

Patients were followed with contrast enhanced MRI imaging during the posttreatment course every 3, 6 and 12 months and every 6 months thereafter. Any enlargement of the pretreatment volume was screened for the occurrence of a RN; the diagnosis was made according to the clinical and radiological course. Treatment was predominantly conservative including dexamethasone and bevacizumab, with surgical debulking indicated only in very large and space occupying lesions.

Data statistical analysis was processed using IBM SPSS Statistics (v.26.0 for Mac OS. Armonk, NY: IBM Corp.). The confidence interval and α were defined as 95%. A normal distribution of scale parameters was checked by the Kolmogorov–Smirnov test and histograms. Mann–Whitney U-test for ranked and scale parameters lacking normal distribution was used. Chi^2^-test comparing two binominal parameters was applied. The multivariate analysis was performed according to terms of Cox regression with occurrence of radiation necrosis as a dependent parameter. The initial model was established for all tumors of our series, where the following variables were included: age, gender, prior radiation therapy, maximal tumor diameter, maximal diameter of the surrounding edema, dose of the radiosurgery, previous resection, amount of dexamethasone and multilocular location.

We stratified eligible tumors into two groups. Meningiomas and VS were classified together as group 1 because of their comparable generally low-malignant clinical course and their extra axial growth and were compared with metastases (group 2). The equal number of RN in both groups allowed a case-matching analysis using the tumor diameter with a 3 mm (+/−) tolerance.

A multivariate model, which includes this categorization as a new variable, was processed. Additionally, a separate Cox regression model with initial independent parameters for each group was established. To eliminate the influence of tumor dignity (BM vs. VS/Meningioma), we matched tumors of these two groups considering maximal tumor diameter (automated processing, tolerance ±3 mm, 1 to 1, *n* = 322) and established re-evaluated Cox regression model with initial variables.

## 3. Results

This retrospective analysis included 388 patients. The gender distribution favored women (217 cases, 55.9%). The median age was 59 years (range 7–91 years). The most frequent tumor type treated were BM with 183 cases (47.2%), followed by VS with 125 cases (32.2%) and 52 cases of meningiomas (13.4%). Furthermore, ependymomas and glomus tumors accounted for 4.1% and glioma cases for another 3.1%. In the gliomas, SRS was indicated in case of small and well delineated recurrent gliomas after previous conventional external beam radiotherapy [17,18].

Metastases are further subclassified based on their primary (Table 1).

The largest mean diameter on axial imaging of all tumors was 16.3 ± 6.1 mm mean (range 2.7–41). The median applied radiation dose was 16 Gy (IqR 14–25, range 12–28). The median cumulative amount of dexamethasone administered was 120 mg (IqR 72–120).

Most of the cases had no previous radiation therapy to the brain (314 case, 81.1%). However, 37 patients (9.6%) had already undergone a previous cranial radiotherapy, 30 patients (7.8%) had 2 previous cerebral radiation therapies and a subset of 6 patients (1.5%) had already had 3 or more previous irradiation therapies to the brain. In about three quarters of the cases (296 patients, 76.9%) radiosurgery was chosen as primary treatment, whereas in the remaining 89 patients (23.1%) a previous surgical intervention was performed.

The median follow-up was 24 months (IqR 6–62, range 0–192 months). RN occurred during this time in 61 patients (15.7%) of the cohort. Mean time to RN was 8 months (range 1–41 months). About half of the patients (*n* = 27/61; 47%) developed a symptomatic RN; the other half was limited to radiological findings.

Twenty-seven (43%) of the RNs required treatment. Conservative treatment (dexamethasone or Bevacizumab) was administered in most cases (*n* = 23), two cases required surgical decompression. The remaining two patients would have necessitated a treatment due to symptoms, but because of the clinical poor condition no further treatment was administered and they received best supportive care.

The incidence of RN as well as the mean diameter and median dose among the most common tumor entities in this series is shown in Table 2.

Both a larger tumor diameter and a higher radiation dose at the 80% isodose were associated with a significantly higher risk of developing a RN in this series in Cox regression. The hazard ratio (HR) for the diameter was 1.065 (Confidence Interval (CI) 1.007–1.127, *p* = 0.028). The HR for SRS dose was 1.302 (CI 1.152–1.472, *p* < 0.001). No significant correlation was shown for gender, previous SRS or resection, surrounding edema, dose of dexamethasone administered or multilocular tumor extension and RN development in Cox regression. Age could only be associated with a significantly increased risk for RN only for metastases (HR 1.066, CI 1.000–1.137, *p* = 0.049).

In univariate analysis of group 1 (VS and meningiomas) and group 2 (BM), BM showed significantly larger edema (*p* = 0.017) and higher doses applied (*p* < 0.001), but a significantly smaller tumor diameter (*p* = 0.039). Furthermore, the number of patients with metastases that had already undergone SRS was significantly higher (*p* = 0.029). No significant differences were found concerning age, previous resections and amount of dexamethasone between the groups. Cox regression of group 1 and 2 showed a significantly higher risk to develop a RN in group 1 (*p* = 0.002).

Due to the similar number of cases between groups 1 and 2, case matching was performed considering the tumor diameter with a tolerance of 3 mm. Three hundred and twenty-two patients could be included. The performed Cox regression considering matching showed a significance concerning tumor diameter (*p* = 0.013) and radiation dose (*p* < 0.001) in relation to the development of RN. No significant differences could be demonstrated with regard to gender, previous resections, age, prior radiation, surrounding edema and multilocular location in the matched Cox regression.

To account for dose conformity and heterogeneity we retrieved also these parameters and ended up with 117 patients of the whole cohort, where conformity indices (CI) and heterogeneity indices (HI) were available. In this subset of patients, no differences could be detected regarding the rate of RNs neither for the CI (*p* = 0.673,), nor for the HI (*p* = 0.111).

## 4. Discussion

Radiosurgery is a technique widely used in the field of neuro-oncology and RN constitutes an important complication with little known common risk factors that may predict its occurrence. Because of the potential neurologic deficits [19] due to radiation necrosis and the associated impairment in quality of life, identification of patients at increased risk is essential. In our series, 15.7% of patients developed radiation necrosis. This underlines the need to explicitly inform patients of possibility of further medical treatment or surgical resection.

A larger axial diameter of tumors was shown to be an important risk factor for RN in this study with a HR of 1.065 per mm diameter. This implicates an increased risk by 65% to develop a RN when comparing a 10 mm to a 20 mm diameter tumor. Larger volume is consistent with reports in the literature as a risk factor for RN [10,20,21]. However, to the best of our knowledge, this study is the first to allow a quickly applicable comparison regarding the risk of radiation necrosis between different cases applicable to multiple tumor types. In clinical practice, this could implicate that other treatment options, such as multiple SRS sessions or surgical resection, should be encouraged for larger neoplasms.

Furthermore, a clear and significant relationship between a higher dose and the occurrence of RN could be identified. This is also consistent with reports from the literature [22,23]. The HR of 1.302 encountered in this study allows physicians to assess the risk for RN and consider other treatment options for tumors that necessitate a higher irradiation dose. The HR of 1.302 implies an increased risk of more than 180% between 14 Gy (median dose for meningiomas) and 20 Gy (median dose for metastases).

Previous studies discussed a possible correlation of higher HI and CI in the prevention of radiation necrosis following intensity modulated radiotherapy techniques [24,25] In our study reporting on conventional SRS techniques, the HI and CI could be determined in 117 of 388 patients only, since the other patients were planned with a planning program before 2007, from which the required data can no longer be determined retrospectively. Within the determined ranges of homogeneity and conformity indices, however, we could not evidence a statistically significant influence of HI and CI on the incidence of radiation necrosis in our cohort of investigated patients.

To the best of our knowledge, this study was the first to demonstrate a correlation between advancing age and the occurrence of RN in brain metastases. This has important clinical relevance, as elderly patients suffer an increased risk to develop malignant neoplasms [26] and might be withheld from microsurgical resection of metastases due to comorbidities [27]. The risk of developing a RN is increased by 132% for a 20 year difference in age. Although stereotactic radiosurgery is a widely used treatment for intracranial tumors in elderly patients [28,29,30] with excellent outcomes, they should be made aware of their increased risk of RN and in case of very large metastases, the possibility of resection should be evaluated, taking into account their comorbidities and pre-existing diseases as well as their general condition, in order to achieve the best possible result.

The analyzed meningiomas in this series showed the proportionally highest rate of RN among all tumors. Considering that almost half (40.4%) of the meningiomas were located at the skull base and this location is known to be a protective factor, a closer look at the tumors of other locations should be given in further studies. In the literature, the parasagittal location of meningiomas has already been associated with an increased risk of radiation necrosis and other post-radiosurgical symptoms such as severe edema [8,31,32]. Given that no significant association between radiation necrosis and prior resection was found in this study, the importance of radiosurgery for recurrent meningiomas, especially at the skull base, may increase in future. Nevertheless, for optimal management of patients harboring meningiomas, prospective studies are needed to identify additional risk factors.

In our analysis, Cox regression revealed a significantly higher rate of RN in the group of benign tumors such as VS and meningioma when compared to BMs. A further case-match analysis was conducted according to the maximal tumor diameter. It was confirmed that not only the applied radiation dose, but also the tumor size remained statistically significant for the development of a RN. The higher incidence of a RN in the VS/meningioma-group accounted for the significantly higher volume of these tumors and outweighed the lower radiation dose needed. Therefore, it can be assumed that the type of irradiated tissue did not play a role in the development of RN but only the dosage of radiation and the size of the tumor were responsible factors for the occurrence of RN.

The fact that no significant difference was shown in the cumulative amount of cortisone administered could lead to a reduced amount of cortisone administration to avoid the potentially considerable side effects. Further, no significant correlation between the development of radiation necrosis and gender was found. This is consistent with existing literature [33] and suggests that gender differences, which exist in meningiomas, do not seem to play a role with regard to RN.

Our study has limitations, such as the retrospective study design. Furthermore, this was a single-center study using an institutional standard for SRS technique, planning and administration. Additionally, different tumor types show different sensitivity to radiation therapy. In our cohort only three types of primaries were included, and other, more radioresistant tumors may behave differently. Therefore, data from our cohort may not be generalizable. Our cohort comprises tumors that have rarely been histologically diagnosed. Included tumors have been undoubted for their tissue type. Further, biopsies of a radiation necrosis was never performed, because histological workup of RN can be cumbersome and histological proof is not mandatory in international guidelines [14]. This suggests that the diagnosis of radiation necrosis was made based only on imaging and the clinical course.

## 5. Conclusions

Independent from tumor entity, we found that larger tumor size and higher radiation doses applied were independently associated with an increased risk of development of RN. As a consequence, different treatment options than single stage SRS should be taken into account for large-volume tumors and expected high radiation doses.

## Figures and Tables

**Table 1:** Primary tumor of BM undergoing radiosurgery.

Primary	*N*	Percent of All Tumors
Non-small cell lung carcinoma	64	16.5
Melanoma	37	9.5
Breast carcinoma	26	6.7
Renal cell carcinoma	11	2.8
Cancer of unknown primary	16	4.1
Other	29	7.5

**Table 2:** Incidence of RN as well as diameter, SRS dose and FU between different tumor types. VS vestibular schwannoma, BM brain metastasis.

	VS	BM	Meningioma
Cases	125	183	52
Mean diameter (mm)	16.7 (SD 5.2)	15.6 (SD 6.9)	18.0 (SD 4.9)
Median SRS dose (Gy)	13 (IqR 12–14)	20 (IqR 18–20)	14 (IqR 14–16)
RN	6 (4.8%)	29 (15.8%)	18 (34.6%)
RN months median (months)	10 (IqR 5.25–12)	9 (IqR 6–16)	8 (IqR 6–12)
FU months median (months)	24 (IqR 6–62)	8 (IqR 3.5–24)	34 (IqR 10.5–94)

## Data Availability

Data available on request due to privacy and ethical restrictions.
